# Online focus groups as a tool to collect data in hard-to-include populations: examples from paediatric oncology

**DOI:** 10.1186/1471-2288-9-15

**Published:** 2009-03-03

**Authors:** Kiek Tates, Marieke Zwaanswijk, Roel Otten, Sandra van Dulmen, Peter M Hoogerbrugge, Willem A Kamps, Jozien M Bensing

**Affiliations:** 1 NIVEL (Netherlands Institute for Health Services Research), P.O. Box 1568, 3500 BN Utrecht, the Netherlands; 2 Department of Paediatric Hemato-Oncology, University Medical Centre St Radboud, Nijmegen, the Netherlands; 3 Department of Paediatric Oncology, University Medical Centre Groningen, Groningen, the Netherlands; 4 Department of Clinical and Health Psychology, Faculty of Social Sciences, Utrecht University, the Netherlands

## Abstract

**Background:**

The purpose of this article is to describe and evaluate the methodology of online focus group discussions within the setting of paediatric oncology.

**Methods:**

Qualitative study consisting of separate moderated asynchronous online discussion groups with 7 paediatric cancer patients (aged 8–17), 11 parents, and 18 survivors of childhood cancer (aged 8–17 at diagnosis).

**Results:**

All three participant groups could be actively engaged over a one-week period. Respondents highly valued the flexibility and convenience of logging in at their own time and place to join the discussion. Adolescent patients and survivors emphasized that the anonymity experienced made them feel comfortable to express their views in detail. The findings indicate a strong preference for online group discussions across all participant groups.

**Conclusion:**

The findings show that online focus group methodology is a feasible tool for collecting qualitative data within the setting of paediatric oncology, and may offer new opportunities to collect data in other hard-to-include populations. The evaluations seem to indicate that the online group discussions have given participants an opportunity to articulate their experiences and views in a way they might not have done in a traditional group discussion.

## Background

The world-wide expansion of access to and use of the Internet over the last twdecades has made the Web a prominent mode of communication, permeating people's personal and professional lives. More recently, the Internet is being recognized as a research tool, offering new opportunities for researchers to conduct quantitative and qualitative research. Gaining popularity first in marketing research, and in communication and media studies, Internet-based data collection is receiving increasing attention in the social and health sciences. [1-8] In particular, the Web offers the unique possibility to include participants who are hard to reach using traditional research methods, e.g. due to time constraints, immobility, or active treatment. [7,9-16]

Interest in online focus groups came at a time when researchers were being assailed by recruitment problems, declining response rates, and rapidly raising costs for traditional focus group discussions. Independent of the setting, patient recruitment often is a bottle-neck in research and a considerable number of projects need much more time than planned to obtain a significant number of patients. [17] In this paper, we intend to contribute to this discussion by describing the methods and feasibility of online focus group discussions within the setting of paediatric oncology.

### New modes of focus groups

A traditional face-to-face focus group (TFG) discussion is a form of group interview, which has been a well-established practice for data collection across a range of disciplines since the early part of the 20^th ^century [18-20]. The method is particularly effective for exploring people's opinions and experiences regarding illness and healthcare, both from a consumers' and a providers' perspective. [15,21-23] The appropriateness of this research tool in eliciting children's views on and experiences in health-related matters has been shown extensively [24-28].

Distinctive terminology may be used for denoting online focus group (OFG) discussions: e.g. computer-mediated or Internet-based focus groups, electronic focus groups, chat-based focus groups, or virtual panel discussions. [1,11,15,29-37] All labels refer to research methods that utilize the Internet to unite spatially and possibly temporally separate participants in text-based group discussions, guided by a moderator. In general, the term OFG does *not *refer to chat rooms or open panel discussions where participants are free to log in at any time, and post their comments on a random topic.

OFGs can be conducted in two ways: *synchronously *(in real time) or *asynchronously *(not in real time), or using a combination of both. In the synchronous mode, participants are online simultaneously at a prearranged time, and immediately react to each other's responses as these are received. The asynchronous mode refers to an ongoing website where participants are free to log in during a set period, read each others' contributions and post a comment themselves at a time that is convenient for them, not necessarily when anyone else is participating.

### Benefits online focus groups

Recent studies on the potential benefits of the use of OFGs were remarkably similar, drawing particular attention to: recruitment issues, participant convenience, researcher benefits, and the quality of the data obtained. The most obvious advantage of using online focus group methodology is that it enables access to populations that are hard to include using standard research techniques and that it facilitates dialogue between participants who may not otherwise have spoken with each other [8,9,11,13,16,30,32,38]. The Internet allows new recruitment opportunities for ill or disabled participants, housebound respondents, marginalized populations, and socially or geographically isolated people. Recruitment is a problem associated with TFG and is likely to grow, because of the increasing difficulty of scheduling meetings for busy people. The use of OFG may be an important tool used to circumvent this problem.

A second significant advantage of using OFGs is providing participants a convenient and comfortable way of joining group discussions. [1,3,6,11,14,16,33,39] Unconstrained by place and time, online participants can contribute to the group discussion at their leisure and individual places. A distinctive feature in asynchronous group discussions is that it allows participants to choose their time in answering questions, allowing more time to reflect.

In addition to these advantages for participants, potential researcher benefits have been documented as well. Foremost amongst these advantages of OFGs, are cost and time-savings due to the automatic and accurate capture of the discussion data. [4,6,9,11,12,35] Responses can be transferred directly in a database where they are immediately accessible for analysis, without the need for transcription or editing, enhancing the accuracy of collected data and eliminating transcriber bias. Additional benefits of OFGs include lower recruitment costs compared with TFGs and the absence of logistic and travel expenses.

### Quality of data obtained

Several studies have investigated the comparability of findings gained from TFGs and OFGs. They seem to suggest that the quantity and quality of data obtained online are broadly comparable to those obtained by traditional focus group discussions. [15,33,34,37,39] Online data collection has the added advantage of providing an effective format to collect sensitive or personal health information. Self-disclosure, defined as 'revealing personal information to others', is a key objective in focus groups, and lack of self-disclosure is often listed as a barrier to effective group discussions. The anonymity afforded by online communication is central in the explanation of increased levels of self-disclosure in OFGs. The perceived privacy makes the online environment more conducive to eliciting honest and thoughtful responses. [30,39-43] Furthermore, the visual anonymity provided by online focus groups may reduce the social desirability bias, allowing participants to feel more comfortable to voice their viewpoints. [5,9,40,43] In traditional focus group discussions, participants may feel silenced or intimidated by more talkative participants. An important advantage of the online mode is the greater equality in participation than in traditional groups, providing a more balanced impression of all the viewpoints being expressed in the discussion. [6,9,33,43] Finally, the absence of time-pressure in asynchronous forms of online discussion, allows considered responses that are lengthier and more detailed than those in synchronous or traditional forms [5,40,43]. The aforementioned advantages attributed to conducting group discussions online enhance the accuracy and objectivity of the data obtained, and, consequently, the quality of the data.

### Examples from paediatric oncology

Guidelines in paediatric oncology encourage health providers to share developmentally relevant medical information with young patients and their parents to enable active participation in the decision-making process. [44,45] Unknown, so far, is to what extent this mirrors patients' and parents' preferences. In view of the merits of OFGs as described above, and the awareness that young patients and their parents are hard to include in group discussions, we expected the online mode of focus group discussions the best choice for this population. Children's familiarity with the Internet further pleads in favour of collecting data online. [3,14,16,46,47] So far, the use of online focus groups for collecting research data has not been explored extensively in paediatric health care. [14,16]

We therefore conducted a series of asynchronous online focus group discussions with children in active treatment for paediatric cancer, their parents, and child survivors of paediatric cancer separately, to determine what constitutes good quality of communication in terms of participation and role delineation from their point of view. The descriptive results of these OFGs have been reported in more detail elsewhere. [48] In this paper, will describe the process of conducting asynchronous online focus groups within the setting of paediatric cancer care and focus on participants' evaluations of joining these group discussions online.

## Methods

### Sampling and recruitment

We identified a sample of three separate groups of participants: 8–17 year old patients in active treatment for childhood cancer (diagnosed 6 weeks to 1 year ago); parents of these children; and a group survivors of childhood cancer, who had been 8 to 17 years old at time of diagnosis, and whose treatment had been successfully finished during the preceding five years. Exclusion criteria were insufficient mastery of the Dutch language, a lag in development, treatment for secondary tumours, and being in the palliative phase of treatment (oncologists' evaluations).

To minimize selection bias, patients and their parents were included by consecutive inclusion, based on order of appointment in two Dutch university paediatric oncology wards. Letters describing the study objectives and requesting participation were given to eligible families by their health care provider, either personal or by regular mail. Family members could participate on an individual basis, meaning that not necessarily both parents and child of each participating family were included. Because survivors did not visit the oncological wards on a regular basis, eligible participants were selected from electronic recording systems in both wards, and invited by regular mail.

Written consent was obtained from 13 (41.9%) of the 31 eligible families, and 8 of them (25.8%) actually participated (7 patients and 11 parents). Nineteen (33.9%) out of the 56 approached survivors agreed to participate, and 18 (32.1%) actually did. Separate group discussions were organised for child patients (aged 8 to 11 years), adolescent patients (aged 12 to 17 years), parents and survivors. A letter containing background information, the homepage URL, an individual login name and password was sent to all respondents who consented to participate. For characteristics of participating patients, parents and survivors: see Table 1. Ethical approval for the study was obtained in advance (METc UMCG 2005-050 and METc UMC St Radboud AMO 05/074).

**Table 1:** Characteristics of participants of online focus groups

	Patients N = 7	Parents N = 11	Survivors N = 18
Age: mean (range)	11.6 (8–16)	45.9 (37–72)	15.5 (10–19)
Age at diagnosis: mean (range)	10.4 (8–15)	-	11.6 (8–16)
Male gender: % (N)	42.9% (3)	45.5% (5)	38.9% (7)
Diagnosis: % (N)			
Leukaemia	42.9% (3)	-	55.6% (10)
Brain tumour	28.6% (2)	-	11.1% (2)
Lymphoma	14.3% (1)	-	16.7% (3)
Bone tumour	-	-	16.7% (3)
Soft tissue sarcoma	14.3% (1)	-	-

### Procedures

We opted for moderated, asynchronous online focus group discussions, conducted following recently developed guidelines for online data collection. [1,4-6,30,31,35,49] Precautions were taken to guarantee both the anonymity of the participants and the confidentiality of their contributions to the online discussions. Each participant was given a unique login name and password, with which they could anonymously access their discussion group website during one week, 24 hours a day. All participants were able to view the most recent contributions in their group as well as scroll back to view earlier responses. The only persons who could be identified by name were two researchers (KT and MZ) who acted as moderators. Their role in establishing a creative, synergistic, non-inhibiting environment for discussion online was very similar to what it would have beeen in a TFG, by regularly checking the postings, asking additional questions to clarify participants' views, and encouraging group discussion. [5,6,30,31] Following conventions used in TFGs, a topic guide was developed, questioning participants' experiences, needs and preferences with respect to communication in paediatric oncology. Instead of introducing all questions at the start of the OFGs, the researchers posted one question a day, aiming at optimal group discussion as recommended in previous research [32,35]. The content of the basic questions posed during the first five days was equivalent for the three groups of participants, with the wording being adapted to the age range of the respondents. On the third day, a reminder was sent to those respondents who had not been joining the discussion. During the last two days, the participants were invited to introduce new topics for discussion they considered relevant in communication in paediatric oncology. Questions of the previous days remained open for responses during the whole week.

Since this was a novel technique, after closing of the OFGs, all participants were invited by e-mail to individually evaluate the process of joining this group discussion online, using a brief semi-structured online questionnaire. Areas of evaluation were drawn from the literature on the benefits and challenges of online discussions, including questions regarding: choices between OFG and TFG, perceived benefits of both modes of group discussions, ease of access, duration of the OFGs, and whether or not to introduce all questions at the start of the discussion.

### Technical equipment

The OFGs were based on a Web Browser that could run on an MS Windows/Web server platform and were served from a sub site of the NIVEL website. This browser-based application was developed in cooperation with the NIVEL Knowledge Centre, using HTML (Hypertext Markup Language) to create the discussion boards. The website included an introduction to the study, the aims of the research, and a description of the group discussion rules. When logging in, a password and login name had to be filled in, ensuring authentication of legitimate participants. At each completion, a programmed notification was sent by e-mail to the moderators, informing them that the participant had posted a message in a specified discussion group. The server was continuously serviced and monitored by professional staff.

## Results

The online focus groups generated rich and detailed qualitative data. The content of the focus groups responses have been reported elsewhere. [48] In this paper, we will focus on the methodology of the online focus groups and participants' evaluations of participating in these groups. Children in active treatment for childhood cancer, their parents, as well as survivors could be actively engaged over a week period and expressed multi-faceted views regarding their communication needs and preferences. Many participants returned to previously answered questions and discussions and made new comments. The mean number of postings per participant varied slightly across the three groups: patients posted an average of 7 responses (range 4–11); parents 4 (range 1–8), and survivors 6 (range 1–15) during the first 5 days of the focus group discussions. All respondents who were initially involved were still accessing the website at the end of the study.

### Focus group dynamics

Focus group dynamics varied considerably between the three groups of participants. Younger children tended to direct their comments to the moderators rather than to each other, but always responded very dedicatedly to the moderators' additional questions and probes. The adolescent patients and the survivors developed a far more interactive way of responding to each other, by reacting actively to each others' contributions. Particularly the participants in the survivor group were very supportive towards one another in acknowledging the experiences their fellow participants described. Several survivors expressed the need for further contact with other participants, as expressed in the following quote: *"I would like to keep in touch with some people of the study*". The group dynamic in the parental focus groups appeared to be far less interactive. Parents entered very long and well-considered postings, but did hardly react spontaneously to other parents' comments. As one parent stated: *"It was not really a group discussion, more like answers to statements"*. However, a lot of parents returned to previously answered questions and posted additional information or clarified their contributions in response to the moderators' questions.

### Participants' evaluations

Four of the 7 child patients, all 11 parents, and 16 of the 18 survivors filled in the evaluation questionnaire after closing of the OFGs. Ease of use of the online group discussions was judged to be good by the vast majority of the respondents (24 of 31 respondents), just as most participants positively valued the frequency of one question a day (24 of 31 respondents). In our study we set a time period of 1 week for joining the group discussion. All participants in the patient group were satisfied with this time frame, whereas most survivors and parents would have preferred an extended duration: *"I think there would be more discussion if people had had more time to think about it" *(survivor).

In response to the question whether or not participants would have attended a group discussion on the same topic if offered in a traditional face-to-face format, 11 participants reacted positively (1 patient, 4 parents, 6 survivors), 11 respondents stated that they would not have joined a traditional group discussion (6 parents, 5 survivors), and 9 participants were not sure (3 patients, 1 parent, 5 survivors). When participants were asked which mode of group discussion they would have preferred to join if they would be free to choose, 22 of 31 participants opted for the online format (3 patients, 9 parents, 10 survivors). Only three survivors and two parents expressed a preference for traditional face-to-face focus groups, and one patient and three survivors could not decide. These findings indicate a strong preference for OFGs across all participant groups.

The evaluation highlighted that most respondents had clear and outspoken ideas about the relative pros and cons of online group discussions. Participants who would have preferred a TFG, considered the online mode of discussion less personal compared to face-to-face communication: "*I, personally, also like to see the people I am talking with." *(survivor), and: *"It is also very cosy and you understand each other better than when you have to tell everything on the Internet" *(survivor). A strong and consistent finding that emerged in each group was the advantage of participant convenience attributed to online communication. Most participants expressed great comfort with the flexibility of logging in at their own place and time: *"This way, I don't have to miss out on extras at school" *(survivor), and: *"It is quite a job to plan being from home for part of the day." *(parent).

Another major advantage that emerged from the respondents' evaluations was the potential for greater anonymity in online interactions. Participants indicated that they felt more comfortable and free to express their views, and worried less about what others might think of them: *"The advantage of a conversation on the Internet is that other people can't see how you handle things, emotions and stuff like that" *(patient). Some participants explicitly expressed that the anonymity experienced during the online discussions stimulated their self -disclosure: *"It is sometimes difficult thinking about hard times you have had in the past. With strangers it is even more difficult to find the right words. On the Internet it is easier to be yourself." *(survivor). Another survivor stated in addition: *"It is hard to tell your story to people you don't know when you are sitting opposite them. I am also a bit shy, so if I had to tell it in a group, I would not tell as much as I would do in this study on the Internet"*. These evaluations seem to indicate that the online group discussions have given participants an opportunity to articulate their experiences and views in a way that they might not have done in a traditional group discussion.

## Discussion

### Benefits online focus groups

This study is, to our knowledge, the first to conduct online focus group discussions within the setting of paediatric oncology. Despite the pervasiveness of Internet access and children's preference for this new medium, researchers have largely neglected its use for collecting data in health care. [14,16,47] The results show that OFGs are a feasible alternative method of obtaining qualitative information from respondents who, by a large majority, would not otherwise have joined our study. Children in active treatment for childhood cancer, their parents, as well as survivors could be actively engaged over a week period and provided balanced feedback about the relative pros and cons of online group discussions.

The asynchronicity of the OFGs provided several advantages. Respondents highly valued the flexibility and convenience of logging in at their own place and time. The format permitted respondents a concomitant increased amount of time for reflection, to respond at length, and the opportunity to change or nuance their opinion.

Our findings also confirmed the importance of anonymity offered by the online environment in stimulating self-disclosure. [1,14,16,39-43] In their evaluations, adolescent patients and survivors emphasized that the anonymity experienced during OFGs made them feel comfortable to express their views in more detail, without worrying about the immediate responses from others. This was reflected in the high equality of participation and the diversity in the responses within each OFG, enhancing the accuracy and objectivity of the data obtained, and, consequently, the quality of the data. [6,9,33,43] These benefits of discussing anonymously are reported to be more sizeable for personal or sensitive topics that are troublesome to handle in a face-to-face environment [14,16,31,39].

The respondents provided thoughtful and often supportive comments. This may be due to respondents' strong commitment to the topic under discussion, as is evident from the subsequent quotes: *"I hope that, with this study, concrete plans can be made how to talk with children with cancer in the future"*. (survivor), and: *"This study is very important, I think, and a good initiative! I would like to cooperate in any follow-up study." *(survivor).

The benefits for the researcher that were realized in terms of time and cost savings due to the automatic capture of the discussion data, while not of the same order as improved quality of the data obtained, were by no means insignificant.

### Limitations

A serious concern associated with online data collection is selection bias, due to non-representativeness of the sample and self-selection of participants. Limited computer access or computer-illiteracy may restrict participation, creating a digital divide between the 'have nets' and the 'have nots', thus leading to age or socio-economic status sampling bias [2-6,41,50]. Self-selection of participants from a non-random pool of Internet users may additionally decrease the external validity.

Like traditional focus groups, however, the aim of our study was to achieve a depth of understanding rather than generalization. In order to avoid the pitfall of selection bias, consecutive inclusion of participants was conducted instead of relying on self-selection through the Internet, thereby increasing the representativeness of the sample, and ultimately, the validity of the results [4-6]. Furthermore, no differences could be detected for responding and non-responding participants. [48]

The number of participants might be another limitation. In the present study only four children and three adolescents in active treatment participated. Although this is not unusual in studies of this nature, it is considered to be the minimum group size for a focus group [24,25,28]. Our overall response was 25.8% for eligible families and 32.1% for survivors. We consider this an adequate response rate, in view of the difficult situation parents and children in active treatment for paediatric cancer are faced with. In two of the families that did not participate despite their initial consent, the child's physical condition had deteriorated dramatically at the start of the OFGs. However, data for comparison are missing, since web-based researchers have not (yet) consistently reported response rates [2,51].

A final shortcoming is that we could not check whether respondents logged in without contributing to the discussion. It would have been helpful to know if participants were accessing and reading the discussions (so-called lurking), or were not entering the focus group website at all. However, the software did not allow for recording of the date and time of logins of non-responding participants.

### Unanswered questions and future research

Probably one of the most widely cited disadvantages to using online group discussions is the reported lack of nonverbal signals. [1,5,6,30,31] The conclusions regarding the loss of these cues in online communication have been very mixed, both with positive and negative connotations. A potential disadvantage of lack of nonverbal cues is the less personal nature of the medium and a heightened potential for misinterpretation of the written communication, leading to the assumption that group dynamic is negatively impacted in an online environment. Other studies emphasize that although the dynamics of communicating in OFGs may differ from traditional forms, there is no conclusive evidence that the Internet indeed is an impoverished or impersonal environment. [1,34,35] In sum, the relative feasibility and reliability of the different modes of focus group discussions still need further exploration. A systematic comparison of both the process and the outcomes of each format will allow researchers to apply the appropriate focus group design, depending on the issues at stake and the purposes aimed at.

Another question that deserves further investigation is that of respondents' preferences for different modes of group discussions. Participants' evaluations of the research method employed may have major implications for their willingness to disclose personal information and to participate in future (health services) research. An intriguing question is whether online research methodologies that relate more closely to the needs of participants are prerequisite to involve a new generation of potential respondents. [14,16]

## Conclusion

Our findings emphasize that online focus groups have considerable potential for gathering high quality data within a relatively short period of time from respondents who are unable or unwilling to engage in traditional group discussions. As such, the methodology offers access to respondents from understudied or marginalized populations that were previously hard to include. We would not wish to argue that online focus groups should totally replace their traditional counterpart, but we anticipate the online format to become a valued option in the researcher's toolbox.

## Competing interests

The authors declare that they have no competing interests.

## Authors' contributions

KT and JB conceived the study and developed its design. KT, MZ and RO developed the focus groups websites, and KT and MZ functioned as moderators in the online focus groups. KT coordinated the research team and drafted the manuscript. The other authors, MZ, RO, SvD, PH, WK, and JB, have been involved in critically revising the manuscript, and have read and approved the final manuscript.

## Pre-publication history

The pre-publication history for this paper can be accessed here:

http://www.biomedcentral.com/1471-2288/9/15/prepub
